# Coherent Raman Generation Controlled by Wavefront Shaping

**DOI:** 10.1038/s41598-018-38302-y

**Published:** 2019-02-07

**Authors:** Mariia Shutova, Anton D. Shutov, Alexandra A. Zhdanova, Jonathan V. Thompson, Alexei V. Sokolov

**Affiliations:** Institute for Quantum Science and Engineering, Department of Physics and Astronomy, Texas A&M University, College Station, TX 77843-4242 USA

## Abstract

We investigate the possibility of tailoring coherent Raman generated spectra via adaptive wavefront optimization. Our technique combines a spatial light modulator and a spectrometer providing a feedback loop. The algorithm is capable of controlling the Raman generation, producing broader spectra and an improved overall efficiency, and increasing the intensity of high-order sidebands. Moreover, by wavefront optimization we can extend the generated spectra towards the blue spectral region and increase the total power of generated sidebands. Mutual coherence and equal frequency separation of the multiple Raman sidebands are of interest for the synthesis of ultrashort light pulses with the total spectral bandwidth extending over ultraviolet, visible and near-infrared wavelengths.

## Introduction

Broadband coherent Raman generation results from molecular modulation in Raman-active media^1^. This process provides a promising way towards the synthesis of sub-cycle pulses of coherent light covering the ultraviolet-visible-near infrared range (UV-VIS-NIR)^2–5^. This source is ideal for studying ultrafast processes in atoms and molecules. Moreover, molecular modulation allows for the possibility of sub-cycle pulse shaping for visible wavelengths. This gives a unique opportunity for optical arbitrary waveform generation (OAWG), which stands as a long term goal for physicists since the invention of the laser^6–8^ and is essential for quantum control of chemical reactions^9,10^.

To initiate the coherent Raman generation one needs to prepare a state of molecular coherence in the medium. It can be done using two optical laser fields (pump and Stokes) which frequency difference matches the vibrational frequency of the Raman-active medium. During this process, a vibrational superposition state is created and all the molecules in any small subvolume vibrate in unison. The phases of this vibration correspond to a coherence wave driven by the applied laser fields. The coherent vibrations, in turn, modulate the fields to produce spectral sidebands shifted by the Raman frequency. When the phase-matching conditions are satisfied the Raman generation process goes efficiently and cascades to generate multiple mutually coherent Raman sidebands. These sidebands are equidistant in the frequency domain (separated by the Raman shift) and cover the whole NIR-VIS-UV region. The vibrational coherence wave is then driven by a combination of all fields, applied and generated.

Typically the phase-matching conditions for the process above are relieved in gases in the near-UV, VIS and NIR regions of the spectrum as the chromatic dispersion is negligible for the wavelengths far from electronic and vibrational resonances. The generated Raman spectra can be adjusted to produce ultrashort pulse or tailored for various purposes. For example, Katsuragawa’s group proposed and experimentally demonstrated the possibility of tailoring relative phase relationships among high-order sidebands by using transparent plates with adjustable thickness and position, thereby achieving remarkable spectral control over the sideband generation process^11,12^. At the same time, in highly dispersive media (crystals)^13^ fulfilling the phase-matching conditions is important for producing Raman sidebands efficiently^14–16^. Overall, these conditions depend on factors such as the refractive index at each of the wavelengths involved, the crossing angle between the two input laser beams, and the interaction length in the Raman medium^17^. With multiple laser fields affecting the phase of the molecular coherence, and then interacting with it to produce polarization waves, the phase-matching conditions may become quite complicated. In the cascaded Raman process the crossing angle between pump and Stokes affects the efficiency and direction of the generated anti-Stokes sideband (AS1); then, the AS1 angle affects AS2 and so on. Therefore, when a high-order sideband is generated, the generation process involves a range of crossing angles, which becomes difficult to optimize simultaneously. In essence, this work addresses the optimization problem mentioned above. As a result we show that optimal generation can be achieved not with a single one, but with a tailored set of $\overrightarrow{k}$-vector components/angles within the adaptively shaped pump beam.

Previously Zhi *et al*. theoretically showed that the crossing angle variations are expected to alter the phase-matching conditions and cause a spectral shift of the generated AS sidebands^17^. Liu *et al*. experimentally showed that the phase-matching angle affects the center wavelength of the individual sidebands^14^. Shon *et al*.^18^ theoretically showed the possibility to tune phase-matching conditions to a different regimes for solid hydrogen. By optimizing the crossing angle between input fields and the interaction length, the input field can be converted either to broadband sidebands generation or to a single high-order sideband. Above works indicate that the phase-matching conditions for noncollinear cascaded Raman generation in highly dispersive media are nontrivial and need further theoretical and experimental study.

With the development of liquid crystal based phase-only spatial light modulators (SLMs)^19^ it became possible to realize various adaptive optics algorithms^20,21^ that help to focus light through turbid media^22–24^, to tailor spectrum of supercontinuum generated in a sapphire plate^25^, to enhance Raman scattering signal^26^, or to improve contrast of coherent anti-Stokes Raman scattering (CARS) microscopy^27^. In addition, Thompson *et al*. showed^28^ theoretically and experimentally that the efficiency of second harmonic generation can be enhanced by using wavefront correction algorithms. In the work of Tzang *et al*.^29^ the authors demonstrated the ability of adaptive optics to control the stimulated Raman scattering (SRS) and four wave mixing (FWM) processes in multimode fiber. They use wavefront shaping of input beams to control the superposition of modes coupled into the fiber. This allows them to manipulate the spectra and intensities of the generated sidebands, i.e. suppressing, enhancing or shifting them.

We extend the above ideas and develop an approach for the optimization of coherent Raman generation with an adaptive wavefront correction. We apply the continuous sequential algorithm with spiral out geometry^30^ to one of the optical fields that drive Raman generation. We set the peak intensity within a spectral range covering one higher order sideband (for example AS10) as the target for the optimization algorithm. As a result of this optimization, accomplished by adjusting the beam profile, we increase the intensity of all high-order Raman sidebands and extend the total bandwidth of the generated spectrum. Broad coherent Raman spectrum is of use for ultrashort pulse production, which can be done by overlapping the generated sidebands and adjusting their relative phases to compensate for delays gained as a result of the dispersion^16,31–34^. Spectral bandwidth in this case defines the resultant length of the produced ultrashort pulse.

## Results and Discussion

### Extending the spectral bandwidth

**Figure 1:**
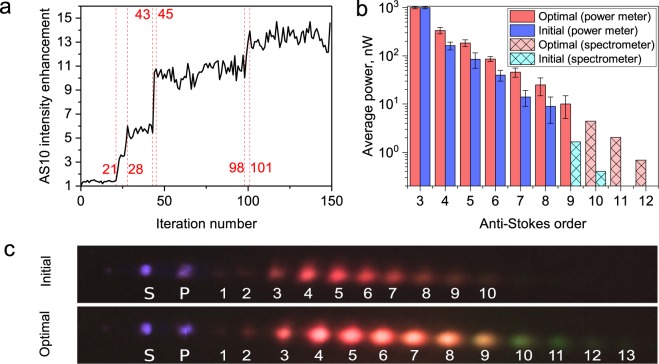
(**a**) AS10 intensity signal detected after every iteration during the optimization process. The graph is normalized by the initial (not shaped) value of the intensity. Red dashed lines show the iterations with the highest increase of intensity on the spectrometer. (**b**) Average power of sidebands with (blue columns) and without (red columns) wavefront shaping, measured with a photodiode power meter. Initial AS9, as well as all of AS10-AS12 powers were below the sensitivity limit of the power meter. Solid columns represent the data taken with the photodiode sensor (power meter) and the plaid columns represent the integrated spectra taken with the calibrated spectrometer. The spectra were integrated over each individual sideband. (**c**) Picture of the row of generated sidebands on a white screen. Top - before optimization, bottom - after optimization; P - pump beam; S - Stokes beam; numbers 1–13 denote orders of generated AS.

In this set of experiments, we generate multiple Raman sidebands using two chirped femtosecond laser pulses. We can set the algorithm to increase/suppress the peak intensity of any chosen spectral region. We pick the spectral region so that the center wavelength of the desired sideband will fall within this region (see “Methods” section for the experimental details). We choose AS10 (570 nm), because it is the highest order sideband generated under our experimental conditions. We set the adaptive algorithm to maximize the peak intensity in the spectral region of 550–600 nm. The ratio of current signal to the initial signal on the spectrometer for each iteration is depicted in Fig. 1a. Each iteration corresponds to the phase optimization of one particular pixel on the SLM array. Several sharp leaps on the graph show that a significant intensity enhancement corresponds to the phase optimization of several individual pixels on the array. Sharp leaps occur due to the phase difference between certain neighboring pixels, which may drastically alter the resultant beam profile (refer to “Methods” section for the details of the optimization algorithm). After optimization, AS10’s intensity is enhanced by a factor of 13 (150^*th*^ iteration).

Albeit we can select various spectral regions for any optimization goals affects all sidebands and not only selected one. We cannot enhance or suppress one sideband without affecting the others; on the contrary, the optimization affects Raman generation overall.

To confirm the overall enhancement we measure the average power of each sideband (Fig. 1b). The optimized total power is ~20% higher than the initial. We see that the power of AS4–AS10 (675-570 nm) sidebands has increased, moreover, the two new higher order sidebands AS11 (558 nm) and AS12 (548 nm) have emerged. Figure 1c shows the comparison of two generated spectra before and after optimization; the spectral broadening and overall brightness increase can be easily seen by eye.

**Figure 2:**
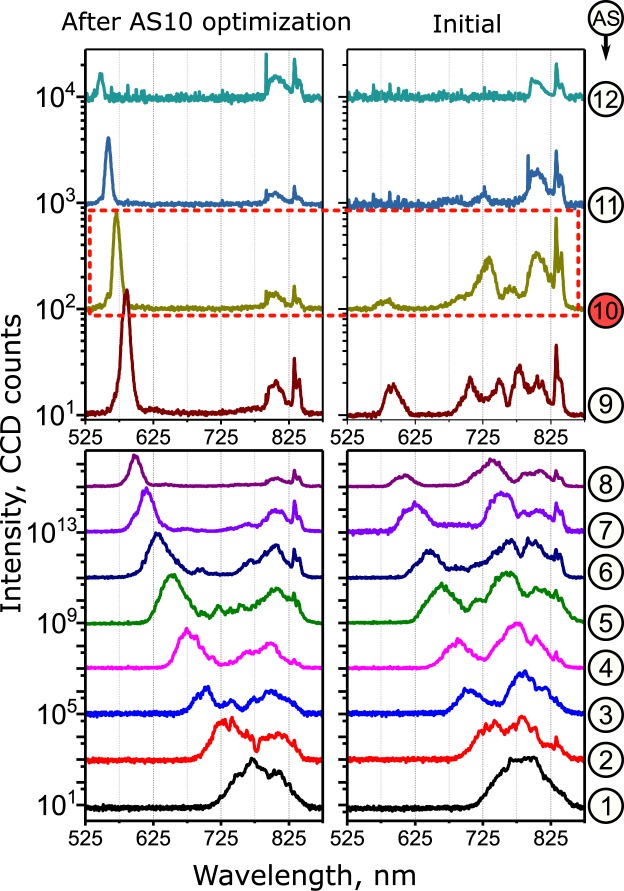
Spectra of the generated Raman sidebands before (right) and after (left) optimization, with the goal to maximize intensity of AS10 (highlighted with red dashed rectangle). Numbers on the right correspond to the order of the anti-Stokes sideband (AS1–AS12). Each individual sideband is spatially separated from the others and focused tightly into the calibrated spectrometer with the help of spatio-spectral filter (see “Methods” for the experimental details). The multiple measured spectra are plotted with a 20 dB (bottom) and 10 dB (top) offset.

Figure 2 shows that after the optimization of AS10, the Raman generation spectra has changed significantly. The linear and nonlinear parasitic scattering near the fundamental laser wavelength has decreased and the center wavelengths of mid- and high- order sidebands have changed (for the optimization of AS5, AS7 and AS10 on a linear scale see Fig. S1 of Supplementary materials).

### Interpretation and simulation

**Figure 3:**
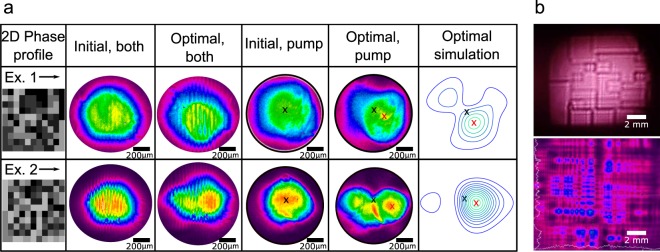
(**a**) SLM phase-masks for the two representative examples of optimization with different beam alignments. Top row - Example 1 (corresponds to the data presented in Fig. 1), bottom row - Example 2. Columns show the spatial phase profile after optimization; beam profiles before and after optimization (the images correspond to beam profiles on the surface of the crystal, ~2.5 cm before the focal plane of the focusing lens). “Both” denotes the profile of overlapped pump and Stokes beams. Contour graphs depict simulated beam profiles at the focal plane of the focusing lens after optimization. Black and red X-es indicate the beam intensity maxima before and after optimization correspondingly. The pump-Stokes crossing angle after optimization is decreasing for Ex.1 by ~0.04°, for Ex.2 by ~0.1°. In addition black X-es indicate the center of Stokes beam in all cases. Sizes are not to scale. (**b**) An example of optimized beam profile as seen on the white screen (top) and as measured by the beam profiler (bottom) in the near-field zone of the SLM.

Figure 3a shows that the adaptive optics algorithm finds the appropriate phase and intensity beam profile to optimize the conditions for the high-order sideband generation. This phase-intensity profile affects the experimental system in several ways.

First, it affects the beam shape and position at the nonlinear interaction area. To quantify this effect we can roughly estimate the beam profile numerically, using Fourier optics. For this, we perform a simulation with PROPER Matlab libraries^35^, based on Fourier transform algorithms^36^. The Gaussian profile with the phase-correction (obtained experimentally, Fig. 3a, first column) undergoes the focusing with the lens. Last column in the Fig. 3a shows the obtained beam profile at the imaging plane (the focal plane of the lens). Although, these simulations come with some caveats because the wavefront distortion added by the SLM smears out the Gaussian mode of light, so that the beam profile varies with the propagation distance. Fourier optics gives us information about the distorted beam profile only at the focal plane. However, we are interested in the imaging plane located 2.5 cm before the focal plane (2 and 3 columns of the Fig. 3a, due to location of the nonlinear interaction area in the experiment). Nevertheless, we see similarities in the simulated (4 column) and experimentally obtained beam profiles (3 column), which adds to the understanding of the effect produced by generated phase profiles. On the other hand, the spatial structure of the generated AS beams does not become complex after the optimization (see Fig. S2 of Supplementary materials), which is a benefit for ultrashort pulse synthesis.

Second, in the most of the experiments, the center wavelength of mid- and high-order sidebands is shifted after the optimization (see Table 1). This serves as a good indicator of the crossing angle change^14,17^. To fully understand the pump-Stokes “crossing configuration” we consider a typical example of the beam profile in the near-field region of SLM (Fig. 3b). It shows the overall complicated picture of the pump beam wavefront, where each intensity maximum has a different phase. This picture represents the space of multiple pump wave vectors (${\overrightarrow{k}}_{pi}$), each of these vectors contributes to the final superposition (${\overrightarrow{k}}_{p}={\sum }_{i=1}^{n}{\overrightarrow{k}}_{pi}$) and affects the final phase-matching for generated sidebands^18^.

**Table 1:** Experimentally measured center wavelengths of generated Raman sidebands AS3–AS12 before and after optimization.

AS order	AS3	AS4	AS5	AS6	AS7	AS8	AS9	AS10	AS11	AS12
Before optimization, wavelength, nm	710	687	665	646	624	611	592	583	—	—
After optimization, wavelength, nm	706	675	653	629	615	596	586	570	558	548

To track capabilities of the adaptive algorithm to change the phase-matching picture, we need to estimate the overall contribution of each small pixel into the final ${\overrightarrow{k}}_{p}$ state, and the role this ${\overrightarrow{k}}_{p}$ plays in generation of n-th AS. This problem is very complex, not only because the phase matching conditions for n-th AS depend on, (n-1)-th AS, but also because of the optimization process is different for each experiment. It depends on the sideband chosen for the optimization and on the experimental alignment.

Nevertheless, we can estimate the crossing angle change for the AS1 generation (the simplest case, dependence only on one pump-Stokes beams orientation). The phase-matching conditions can be seen from the equation for the phase mismatch factor for Raman generation^37^:

1 $$M=sin{c}^{2}[({k}_{R}-{k}_{opt})\times L/2],$$

where (*k*_*R*_ − *k*_*opt*_) stands for the wave vector mismatch, *L* = 500 *μm* - the crystal thickness, *k*_*R*_ - the wave vector modulus of the generated Raman sideband, *k*_*opt*_ - the wave vector modulus of the generated Raman sideband calculated from the geometrical representation of wave vectors; for example, for AS1:

2 $${k}_{opt}={[4{k}_{p}^{2}+{k}_{S}^{2}-4{k}_{p}{k}_{s}cos\theta ]}^{\mathrm{1/2}},$$

where *k*_*p*_ and *k*_*S*_ - are the moduli of the wave vectors for the pump and Stokes fields correspondingly. The mismatch factor characterizes the efficiency of Raman anti-Stokes wavelength generation. Ideal phase matching (*k*_*R*_ − *k*_*opt*_) = 0 and *M* = 1 in (1) ensures a fixed relative phase between generated anti-Stokes wave and nonlinear polarization and efficient energy extraction from input fields. On the other hand when 0 < *M* < 1 the process goes less efficiently.

To estimate the AS1 generation efficiency dependence on the phase-matching angle change quantitatively, we compare the experimentally detected beam positions before and after the optimization by looking at the beam intensity maximum. Using ray optics we calculate that the propagation angle change for various optimizations (different experiments) can be as large as ~0.1°. Therefore, the difference in the phase matching factor for AS1 for the case *M*(2.3°) = 0.76, *M*(2.4°) = 0.91, (the Sellmeier equation for the refractive index was taken from^38^) is equal to *δM* = 0.15. Moreover, the direction of optimized *k*_*AS*1,*opt*_ is changed in comparison with non optimized *k*_*AS*1_.

Next step will be to estimate AS2 generation change after the optimization, because of *k*_*p*_ and *k*_*AS*1_ changes, and so on for AS3, AS4 - AS12. This procedure describes step by step the changes produced by wavefront optimization, that significantly affect the high-order sidebands generation. The overall picture ends up being quite complicated. Figure 3b shows, in essence, that the generation is optimized by a (non-trivial) combination of pump *k*-vectors, but not just one optimal *k*-vector.

## Conclusion

We demonstrate the ability of adaptive optics to increase the intensity of Raman sidebands using the wavefront optimization. We show that the same technique extends the total spectral bandwidth of Raman sideband generation by phase-only corrections to the pump beam profile. The optimization algorithm finds the phase-intensity beam profile which is able to improve the coherent Raman generation. We find that the center wavelengths of the generated sidebands become shifted after the optimization, which can be attributed to the change in the beams crossing angles. We describe the step by step process of phase-matching optimization. The obtained extended spectral bandwidth (and enhanced Raman sidebands) at given total average power is advantageous for generating shorter and more energetic sub-cycle light pulses.

## Methods

### Optical layout

**Figure 4:**
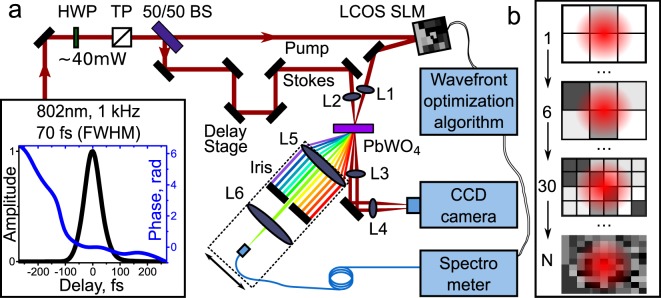
(**a**) Experimental setup for optimized Raman sideband generation with feedback-based wavefront shaping algorithm. HWP - half-wave plate, TP - thin polarizer, BS - beam splitter, L1 - L6 are focusing plano-convex lenses. A sample pulse retrieved via SHG FROG is shown in the inset. Optics in dashed square (L5, L6, Iris, spectrometer head) can be moved along the generated Raman spectra for the single sideband spectrum detection. (**b**) Shaping algorithm; the numbers indicate the iteration and the pictures show the SLM screen with the Gaussian beam profile, square blocks depict optimization pixels, and gray color code shows phase variation of phase profile of the beam.

Our setup is based on the idea of driving molecular vibrations with a pair of time-delayed linearly chirped pulses^39^. The experimental layout is depicted in Fig. 4a. We use a Ti:sapphire femtosecond oscillator + amplifier laser system (Coherent Micra, Legend) with Fourier transform limited ~35 fs, 3.7 mJ laser pulse (1 kHz repetition rate). We stretch the pulse in time up to ~70 fs (spectral width 476 *cm*^−1^) FWHM by offsetting the compressor inside the amplifier from optimal position. By doing this we introduce a linear chirp to the pulse and allow the generation of sidebands due to the excitation of the 325 *cm*^−1^ Raman mode in a PbWO_4_ crystal^39^. The Raman coherence lifetime for this mode is *T* ≈ 2.5 ps^40^, excitation occurs in the transient regime τ_*p*_ ≪ T, where *τ*_*p*_ denotes the time duration of pump pulse^17^.

The chirped pulse then passes through a half-wave plate and a polarizer system for attenuation (down to 40 *μ*J) and more precise polarization control. The pulse is then split by a 50/50 beam splitter. One half of the pulse is used as a pump, the other is used as Stokes. The pump pulse then reflects off of the SLM (LCOS SLM, Hamamatsu X10468-01) and acquires a wavefront modification. The Stokes pulse passes through a retro-reflector on a translator (delay stage) for precise time delay control. Finally, we overlap pump and Stokes pulses in time and space inside the PbWO_4_ Raman crystal. Both beams are loosely focused by 50 cm lenses L1 and L2 behind the crystal at a ~2.5 ± 0.5° angle (the spot size of the beam on the crystal surface is ~600 *μ*m, ~2.5 cm before the focal plane). The average power in each of the two beams incident onto the crystal surface equals to ~13 mW. The pump and Stokes pulses overlap in time and space and produce multiple orders of Raman sidebands separated by the Raman shift. We collimate the produced sidebands by 5 cm plano convex lens L5 and use an iris diaphragm to filter out the particular sideband we chose for optimization. The sidebands are focused with a lens (L6, 10 cm focal length) and coupled into the spectrometer’s input fiber (multimode, 600 *μm* core, numerical aperture 0.22, acceptance angle 12.7°). Convergence angles of the sidebands are small (~0.31°), which ensures good coupling. We measure the average power of the sideband with the photodiode sensor (Ophir PD10-PJ-V2, sensitivity limit - 10 nW), not shown. Lenses L3 and L4 image pump and Stokes beam profiles onto the CCD camera.

### Spatio-spectral filter

Since the phase-matching conditions for cascaded Raman scattering in crystals are non-collinear, each higher-order anti-Stokes frequency is generated at a certain angle to the previous (lower-order) one and all the generated sidebands are spread out in a horizontal plane. For convenience the sidebands are collimated shortly after the crystal with the lens L5 and lower order sidebands are attenuated by a set of neutral density filters (not shown). To measure the spectrum of each individual sideband, we ensure its separation from all other sidebands with an iris diaphragm and focus the separated sideband with the lens L6 into the fiber spectrometer (Ocean Optics USB2000+). Therefore, the spectrum of each sideband were measured separately to ensure no aberrations by centering it on the lenses (spatio-spectral filter is highlighted with the black dashed square in the Fig. 4a). One concern for this configuration is whether the input beam shaping will affect the fiber coupling efficiency. To get reasonable comparison of intensities before and after the optimization, we focus the light tightly into the spectrometer’s fiber (diameter 600 *μm*) to ensure a good, constant coupling efficiency for all spectral components of light.

### Wavefront optimization algorithm

The spectrometer and the phase-only SLM are controlled by an adaptive feedback algorithm running in a closed loop fashion. This algorithm is known as an iterative continuous sequential algorithm with spiral out geometry^30^ (Fig. 4b). Initially, the algorithm splits the SLM screen into blocks of pixels (6 is the minimum number of blocks, Fig. 4b1), and varies the phase applied to the 1st block of pixels from 0 to 2*π* in steps of 2*π*/7. While doing this, the algorithm detects the signal on the spectrometer within a chosen spectral range for each phase value (Fig. 1a). After finishing spectral detection for the 1st block, the algorithm compares the detected spectra, chooses the one that matches with the optimization condition in the best way and memorizes it along with the corresponding phase value for this block. After the 1st block (1st iteration), the algorithm moves to the 2nd block (Fig. 4b6). When all blocks are checked, the algorithm divides current blocks into smaller subblocks (Fig. 4b30) and does the phase variation and spectral detection again^30^. The algorithm can be manually stopped at any point or when the desired result is achieved (Fig. 4bN). The user can choose among several optimization goals, for example: (a) maximize the peak intensity within a certain spectral range; (b) maximize the average intensity within the required spectral range. Any desired spectral range can be specified arbitrarily within the range of the spectrometer.

We have found that careful alignment of the setup is important for our experiments due to the sensitivity of both the wavefront correction technique and cascaded Raman process. We ensure the spatial overlap of the pump and Stokes beams in the crystal and the equality of their diameters and intensities with the beam profiling CCD camera (Spiricon SP620U). We choose intensities that are sufficient for Raman generation while avoiding parasitic effects such as four wave mixing or self-phase modulation. The pump-Stokes frequency difference, their crossing angle, polarizations and beam profiles play an important role in satisfying phase-matching conditions and must be carefully optimized. We measure a temporal chirp and pulse duration with SH-FROG^41^ and detect no spatial chirp in the beam according to the refs^42,43^.

## Supplementary information

Supplementary materials Coherent Raman Generation Controlled by Wavefront Shaping

## Data Availability

All data generated or analyzed during this study are included in this published article.
