# Supplementary material for: Coherent Raman Generation Controlled by Wavefront Shaping
Source: Sci Rep. 2019 Feb 7;9:1565. doi: 10.1038/s41598-018-38302-y (PMC6367464; doi:10.1038/s41598-018-38302-y)
Supplement: Supplementary file 1 — Supplementary materials Coherent Raman Generation Controlled by Wavefront Shaping [file 41598_2018_38302_MOESM1_ESM.pdf]

Supplementary materials
Coherent Raman Generation Controlled by Wavefront Shaping

Mariia Shutova, Anton D. Shutov, Alexandra A. Zhdanova,
Jonathan V. Thompson, Alexei V. Sokolov

December 3, 2018

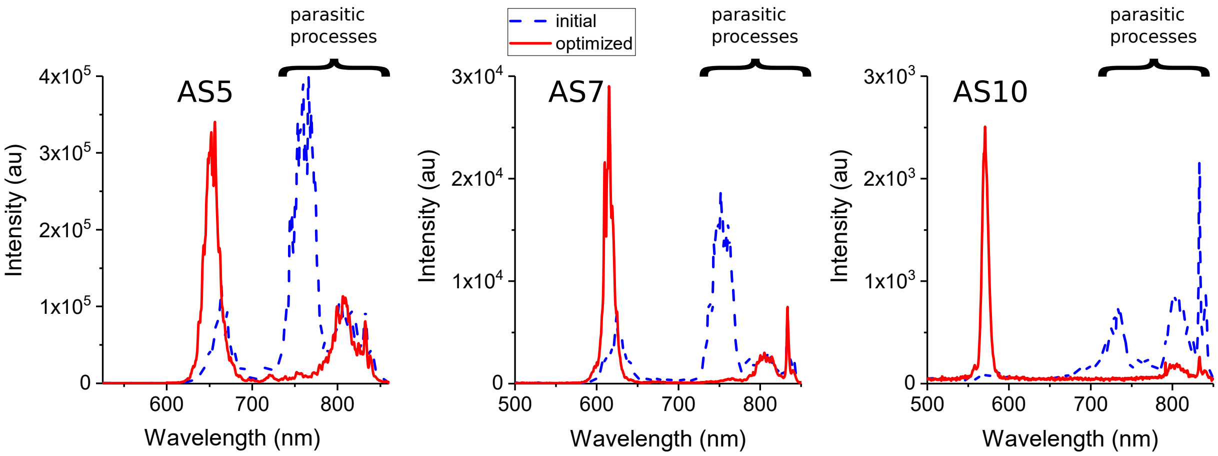

Figure S 1: AS5, AS7 and AS10 spectra before (blue dashed line) and after (red solid line) optimization. The spectra are the same as in Fig.2 of the original manuscript, but on a linear scale. Initial and optimized corresponding sidebands are depicted on the same graph for clarity.

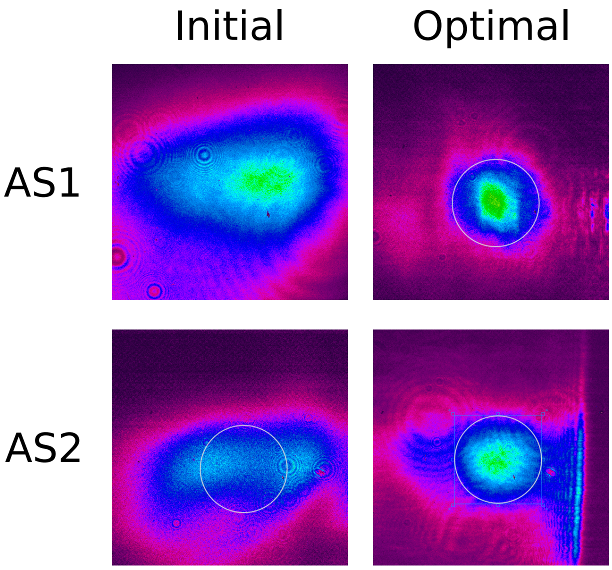

Figure S 2: AS1 and AS2 beam profiles before (initial) and after (optimal) optimization. Elliptical shape of the initial sidebands can be explained by presence of self diffracted sidebands. These sidebands propagate almost collinear to the low order sidebands due to small wavelength difference. As can be seen from the picture, the spatial structure of the generated sidebands is more round close to a Gaussian.
